# An Analysis of the Effect of Skew Rolling Parameters on the Surface Quality of C60 Steel Parts Using Classification Models

**DOI:** 10.3390/ma17215362

**Published:** 2024-11-01

**Authors:** Konrad Lis

**Affiliations:** Department of Metal Forming, Mechanical Engineering Faculty, Lublin University of Technology, 20-618 Lublin, Poland; k.lis@pollub.pl

**Keywords:** skew rolling, C60 steel, roughness, CNC rolling mill, stepped axles and shafts, metal forming, machine learning

## Abstract

This paper presents the experimental and numerical results of a study on producing axisymmetric parts made of the C60-grade steel by skew rolling. The experimental part of this study involved conducting the skew rolling process with varying parameters, including the forming angle *α*, tool angle *θ*, chuck velocity *V_u_*, and reduction ratio *δ*. Their effect on the quality of produced parts was examined and described by the roughness parameter Ra. Numerical calculations involved the use of machine learning models to predict the quality class of produced parts. The highest prediction accuracy of the results was obtained with the random forest and logistic regression models. Metrics such as precision, recall and accuracy were used to evaluate the performance of individual models. Confusion matrices and ROC curves were also employed to illustrate the performance of the classification models. The results of this study will make it possible to prevent the formation of spiral grooves on the circumference of steel parts during the rolling process.

## 1. Introduction

Metal forming is one of the key manufacturing techniques that makes it possible to produce parts and components with complex shapes and high mechanical properties in an efficient and cost-effective way. Given its wide application and capacity for improving the mechanical properties of materials, metal forming plays an important role in many industrial sectors, including automotives, aerospace, construction, energy and heavy industry. When it comes to large-lot production, metal forming is competitive with other manufacturing methods, such as machining, because it ensures a shorter production time as well as reduced material losses. Forming methods are characterised by process complexity and require that process parameters be controlled to achieve the desired quality and properties of finished products. The introduction of advanced techniques for process operation and control along with the use of modern techniques such as machine learning can significantly improve the efficiency and quality of forming processes. The optimisation of these processes will, in turn, allow for the development of more precise production strategies, enabling reduced manufacturing costs.

The application of machine learning methods in cold-rolling stainless steel sheets was studied by Contreras-Fortes et al. [1]. The study used artificial neural networks to predict the mechanical properties of 16 different grades of steel, including austenitic, ferritic and duplex. The following parameters were calculated: the tensile strength (*R_m_*), yield strength (*R_p_*), hardness (*H*), and elongation (*A*). Their values were predicted based on a database containing the chemical properties of the tested materials and the applied cold thickness reduction. The numerical results and the experimental findings showed agreement. High R^2^ ratios were obtained for each tested parameter. The study made use of the SHAP library to determine the effect of individual chemical elements on the obtained mechanical parameters for three steel grades under analysis, i.e., austenitic, ferritic and duplex. Zhao et al. [2] proposed the fusion of random forest and factor analysis (RFFA) algorithms. Such fusion simplifies the model input. The weight matrix is employed, instead of the traditional average method. The refinement of the model resulted in more accurate predictions of mechanical properties, such as yield strength, tensile strength and elongation.

Chemical compositions of 92 steel grades, process settings and steel properties derived from previous rolling operations were used as input data. With the selected hyperparameters, the prediction accuracy was high, exceeding 93%. Li et al. [3] proposed a new deep learning model, obtaining a higher prediction accuracy of the results compared with other models, such as ridge regression, random forest, stepwise regression and support vector machines (SVMs). In effect, the new model was found to be suitable for predicting the mechanical properties of hot-rolled strips, e.g., of carbon and structural steels. Song et al. [4] used machine learning models to obtain high-quality steel products by hot rolling. Artificial neural networks, random forests and support vector machines were used to perform the calculations. The latter model, i.e., the SVM, achieved the best prediction performance, as expressed by the determination coefficient R^2^. Wang et al. [5] predicted the surface quality of steel strips based on the industrial data of hot rolling. Four machine learning algorithms were used, including XGBoost, random forest, support vector machines, and Multi-Layer Perceptron. The XGBoost model trained on the provided test set produced the lowest error indicators (MAE, RMSE), demonstrating its optimal generalisation performance, described by the determination coefficient R^2^.

Idzik et al. [6] used reinforcement learning via Deep Deterministic Policy Gradient in rolling. The proposed model enabled the design of pass schedules in a rolling process of S355 structural steel, so as to achieve the target grain size while at the same time maximising the mechanical properties of the sheet and minimising the energy consumption of the process. The study showed that the model predicted the austenite and ferrite grain sizes with an accuracy of up a few μm. Zhao et al. [7] used neural networks to predict the flatness of cold-rolled strips. Additional algorithms were applied to increase the accuracy of the model (expressed via the coefficient R^2^) and to reduce measurement errors RMSE and MAE. As a result, the proposed model demonstrated higher prediction accuracy and better performance than the generalised model.

It should be emphasised that machine learning can also be used to predict other properties than product quality. Lian et al. [8] applied machine learning to predict forces in the isothermal rolling of titanium–aluminium (TiAl) alloys. A genetic algorithm optimised back propagation neural network was used to that end. The input data for the model were process parameters, including roller temperature, rolling speed, reduction ratio and friction coefficient. The correlation coefficient was over 99%, and the average relative error was 1.6%. Thakur et al. [9] applied machine learning to predict roll force and torque in the plate-rolling of micro-alloyed steel. The results helped to optimise the rolling process. The required microstructure was obtained by increasing the cross-sectional reduction ratio during the finishing tool passes. The mechanical properties were improved, too. Bagheripoor et al. [10] used artificial neural networks to predict force parameters in a rolling process for aluminium sheets. Specifically, the study investigated the roll force and roll torque in this process. The results obtained by the models (for different sets of process variables) were compared with the FEM numerical results of simulations carried out in Abaqus. It was found that with the correctly selected hidden layers and neurons, the models were capable of predicting the rolling process parameters with high accuracy.

The use of machine learning methods can also be applied to other metal forming processes. Trzepiecinski et al. [11] applied these techniques to a single point incremental forming process. They studied the effect of forming parameters on Sa and Sz roughness using artificial neural networks. The roughness was checked in stiffened ribs made in EN AW-7075-T6 and EN AW-2024-T3 Alclad aluminium alloy panels. Möllensiep et al. [12] analysed the process of incremental sheet metal forming using ANNs. The process is still in need of improvement in forming accuracy. The approach used made it possible to predict geometric accuracy and modify the tool path. A reduction in part forming accuracy errors was made. This increased the potential of this process for unit production of products. In the publications presented, machine learning models learn from datasets.

Further development of work on models in metal forming may include the use of input data in the form of physical phenomena. This scheme of operation was applied by Li et al. [13]. They used a modified artificial neural network that applied a physical model as the activation function. They also made modifications to the loss function. This allowed the authors to obtain lower RMSE values (up to two times) compared to the standard artificial neural network. This contributed to an increase in the effectiveness of the fatigue life prediction of welded joints.

In summary, the use of machine learning techniques makes it possible to predict the mechanical properties of formed materials, the surface quality and possible defects of products and force parameters. This applies both to rolling processes, which is the main topic of this publication, but also to other methods, such as sheet metal forming.

### Skew Rolling

The above-presented applications of machine learning algorithms demonstrate the potential of automating metal forming processes. One of the currently developed forming techniques is skew rolling. This process can be used to manufacture elongated axisymmetric parts by three tapered rolls. Pater et al. [14] conducted an experimental study on skew rolling three different preforms made of C45 steel. They obtained satisfactory results for the geometric dimensions of rolled parts. It should be noted that the manufacturing time did not exceed 52 s for the longest part. Skew rolling can also be used to manufacture finished parts, including railcar axles. Pater et al. [15] carried out laboratory tests on forming 42CrMo4 steel railcar axles (in a scale of 1:5) conforming to European and US standards. The examination of the finished parts ruled out the presence of internal cracks. However, spiral grooves were observed on the surface of the products (particularly in the tapered part). It was proposed that the skew rolling process be optimised by conducting it in two stages: first, one end of the workpiece is deformed by the rollers, and then the actual rolling operation is performed. This would help eliminate material waste in the shank part. Xia et al. [16] investigated the effect of skew rolling parameters on the quality of hollow steel axles. The study analysed the causes of surface defects in the form of spiral grooves on the surface of rolled parts. An ANOVA analysis was performed to determine the optimal combination of process parameters affecting the quality of produced parts. Zhang et al. [17] carried out a numerical study to prevent the formation of spiral grooves on the surface of 30CrMoA steel parts. They proposed that the rotational speed of the tools be increased depending on the rolling conditions in order to improve product quality. Wang et al. [18] investigated the quality hollow parts made of 30CrMo steel. It was found that the use of irregular billets could greatly improve the wall thickness quality of formed parts. Wang et al. [19] also conducted fatigue tests on rolled hollow axles made of 30CrMo steel. The tensile and rotational bending tests confirmed that the produced axles had good mechanical properties. The study showed that fatigue cracks originated from the surface of the specimen, most of which were caused by crystal slip, while a small part was caused by inclusion clusters. One of the proposed solutions to improve the skew rolling technique was to increase the number of working tools [20]. The aim of this improvement was to achieve a greater cross-sectional reduction in a single tool pass during rolling. The solution involved using a set of front-tapered rollers to reduce the initial diameter of the workpiece. Later in the process, however, a set of rear rollers would be used to reduce the workpiece to the required geometric dimensions. Cao et al. [21] proposed an alternative method of skew rolling using two tapered rollers. The process was conducted without a workpiece-holding chuck. Two support sleeves were used to maintain the workpiece in the rolling axis. The workpiece was moved as a result of the askew positioning the working tools.

This paper presents the results of a skew rolling process for elongated parts made of the C60-grade steel. A series of experiments was conducted with varying parameters affecting the surface of rolled parts. An analysis was carried out using machine learning techniques in order to select the optimal classification model. The results will help to predict the *Ra* roughness of finished parts, as surface quality may affect further processing, e.g., it may lead to the formation of internal defects during forging.

## 2. Material and Methods

### 2.1. Experiments

**Figure 1:**
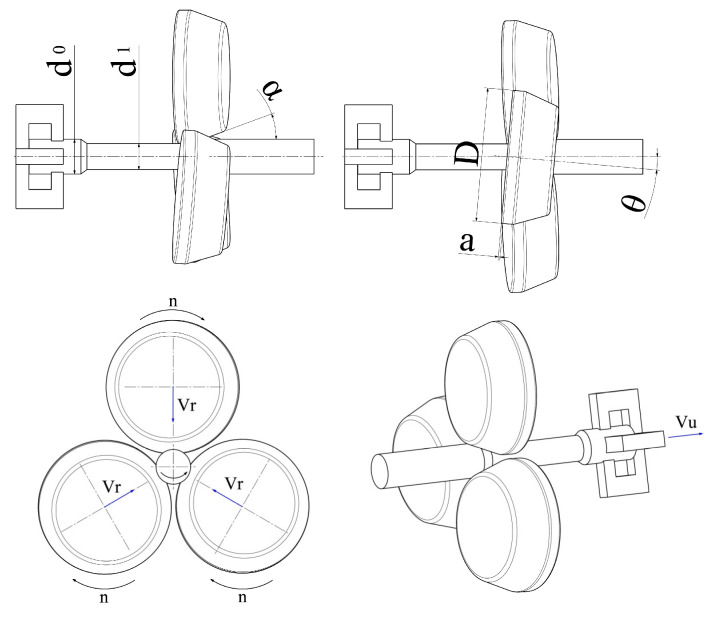
Scheme of a skew rolling process (symbols explained in the text).

The skew rolling process was carried out according to a scheme shown in Figure 1, with the use of tapered rollers described by a forming angle *α* of 15°, 20° and 25°. Each tool was positioned askew to the rolling axis at an angle *θ* of 2.5°, 5° and 7.5°. In addition, each roller was rotated at a constant rate *n* of 60 rpm. In this process, the workpiece was deformed as a result of the synchronised feed motion of the tools and the jaw chuck. In the experiments, the tool velocity *V_r_* was maintained consistently. On the other hand, the chuck, in which one end of the workpiece was fixed, was moving with three different velocities *V_u_*, i.e., 10 mm/s, 20 mm/s and 40 mm/s. During the synchronised tool/chuck motion, the initial diameter *d*_0_ of the workpiece was reduced to a pre-set value *d*_1_. In effect, it was possible to produce different parts, the shape of which resulted from the pre-programmed motion of the rollers and the chuck.

**Figure 2:**
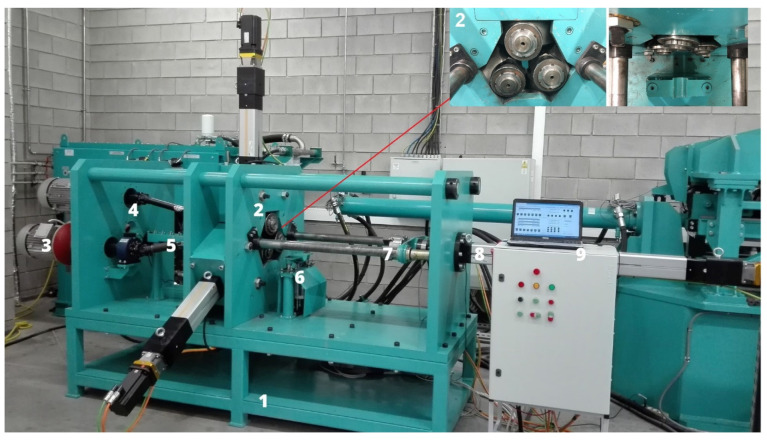
Laboratory skew rolling mill (symbols explained in the text).

The experiments were conducted on a laboratory skew rolling mill [22], shown in Figure 2.

The rolling mill has a modular design. It consists of a support frame (1) on which a plate structure of the rolling mill is placed. The mill’s drive system (3) consists of three electric motors. A powertrain (4) consists of jointed shafts and enables the rotation of tapered rollers that are located in a mill stand (2). The workpiece is mounted in a specially designed support assembly (5). During rolling, the workpiece is moved by a jaw chuck (7), and is supported by a specially designed support subassembly (6). The feed motion of radial cylinders and axial cylinders (8) is synchronised by a programmable automated controller (PAC) (9). The control system, as well as visualisations of process variables, are powered by Codesys. In addition to that, force parameters are recorded in real time during the rolling process. The control application makes it possible to upload a *dxf* file showing the outline of the final product. The file is then converted into a machine code (G-code), enabling the correct movement of the tapered rollers and jaw chuck. In effect, it is possible to roll parts of different shapes by changing the sequence of working tool movements. This makes the process cost-effective, even for unit production.

**Figure 3:**
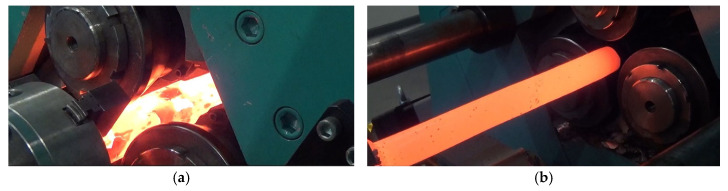

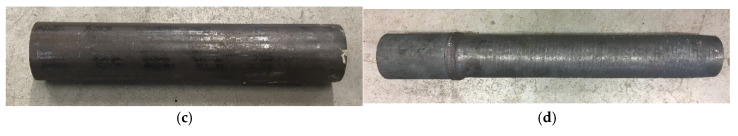
(**a**) Start of rolling, (**b**) end of rolling, (**c**) sample, and (**d**) rolled part.

Samples for the experiments were made of C60-grade of steel. The workpiece had a diameter of 52 mm and a length of 330 mm (Figure 3c). The chemical composition of the material is given in Table 1. Each sample was heated in an electric chamber furnace to a temperature of 1200 °C. After that, they were then mounted in the jaw chuck (Figure 3a) and deformed by skew rolling on the laboratory rolling mill (Figure 3b).

The experiments were conducted according to the settings given in Table 2. The variables were the forming angle *α*, the tool angle *θ* and the axial velocity *V_u_* of the jaw chuck. Their values are given at the beginning of Section 2. The reduction ratio *δ* was also made variable. This parameter describes the cross-sectional reduction and is expressed by the following equation:

(1) $$\delta =\frac{d_{0}}{d_{1}}$$

where *d*_0_—initial diameter of the billet, and *d*_1_—reduced diameter of the rolled part. The following reduction ratio values were used: 1.13, 1.3, and 1.53.

In summary, for one set of tools with a given forming angle *α*, 27 experimental tests had to be carried out. At a given angle *α* of the tapered rollers, the tools were positioned askew to the rolling axis at an angle *θ*. These were equal to 2.5°, 5° and 7.5°. For each of these angles, three jaw chuck velocities were determined, i.e., *V_u_* = 10, 20 and 40 mm/s. Then, for each of these velocities, three different degrees of reduction ratio *δ* were applied, with values of 1.13, 1.3 and 1.53.

The second stage of the experiments involved measuring the surface roughness of rolled parts with Hommel-Etamic’s 3D T8000 RC120-400. This surface roughness tester is a contact probe with a diamond stylus tip. The roughness profile *Ra* was evaluated over a measuring length of 48 mm with a constant speed of 1 mm/s. Measurements were taken several times along the axis of the forgings to obtain repeatable results. The samples were also rotated to allow for the most accurate calculation of *Ra* roughness across the surface of the products.

### 2.2. Numerical Modelling

The numerical analysis involved predicting the surface roughness of steel samples using selected machine learning models. For this purpose, classification models such as logistic regression, random forest, SVC (from the support vector machine group) and XGBoost were employed. The first three models were taken from the scikit-learn library [23], while the last one was obtained from XGBoost [24].

The dataset was created from the obtained results and skew rolling settings. It was divided in a ratio of 80% (training set) to 20% (test set). The input data for the models included the parameters listed in Table 2, i.e., the forming angle *α*, tool angle *θ*, chuck velocity *V_u_* and reduction ratio *δ*. The parameter for the models to predict was the roughness *Ra*. Due to the use of classification in this analysis, the variable *Ra* was divided into three groups. In this way, it was possible to obtain intervals with similar amounts of data and thus avoid an unbalanced division. The target variable was then binarized using the LabelEncoder method, which allowed the data to be used in the numerical analysis. The input variables to the models were also standardised using the StandardScaler method from the scikit-learn library. This was done in accordance with the following relationship:

(2) $$z=\frac{x-\mu }{\sigma }$$

where *x*—experimental value of the input variable, *μ*—arithmetic mean of the input data for a given parameter, and *σ*—standard deviation of the input data for a given parameter. The hyperparameters were selected with BayesSearch imported from the scikit-optimize library. A 3-fold cross-validation on the training dataset was performed in order to avoid overfitting.

Model performance assessment was made with the use of the following metrics: accuracy, precision, recall and *F*_1_ score. Accuracy is defined as the ratio of correct predictions to all predictions. Precision is described by the following equation:

(3) $$P=\frac{T_{p}}{T_{p}+F_{p}},$$

and is defined as the number of true positives (*T_p_*) over the number of true positives plus the number of false positives (*F_p_*). Recall is described by the following equation:

(4) $$R=\frac{T_{p}}{T_{p}+F_{n}},$$

and is defined as the number of true positives (*T_p_*) over the number of true positives plus the number of false negatives (*F_n_*). *F*_1_ score is described by Equation (5):

(5) $$F_{1}=\frac{2T_{p}}{{2T}_{p}+F_{p}+F_{n}},$$

This provides a balanced assessment of model performance, which is expressed as a harmonic mean of precision and recall. Graphical methods such as confusion matrices and ROC curves were also employed to analyse the models.

## 3. Results and Discussion

### 3.1. Experimental Results

**Figure 4:**
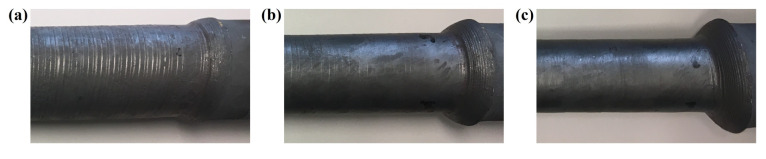
Surface of parts obtained from a rolling process conducted with *α* = 20° (*θ* = 5°) and *V_u_* = 20 mm/s: (**a**) *δ* = 1.13, (**b**) *δ* = 1.3, (**c**) *δ* = 1.53.

The tests conducted according to a specified experimental design made it possible to obtain samples of varying surface quality. A selected few are presented in Figure 4.

**Figure 5:**
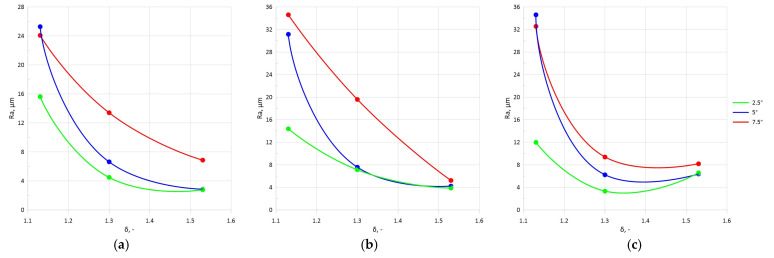
Effect of selected setting parameters on roughness *Ra* at *V_u_ =* 20 mm/s and varying *θ*: (**a**) *α =* 15°, (**b**) α = 20°, (**c**) *α =* 25°.

**Figure 6:**
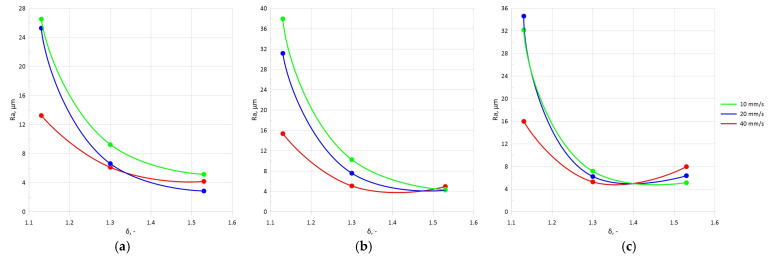
Effect of selected setting parameters on roughness *Ra* at *θ =* 5° and varying *V_u_*: (**a**) *α =* 15°, (**b**) α = 20°, (**c**) *α =* 25°.

The surface layer examination showed that an increase in the forming angle *α* caused an increase in the roughness *Ra*, leading to lower surface quality of the rolled parts. The largest *Ra* increase amounted to 25% and was observed when the *α* angle was increased from 15° to 20°. An increase in the tool angle *θ* also resulted in a higher surface roughness of the steel samples. The largest increase of approximately 56% was observed when the *θ* angle was increased from 2.5° to 5°. The surface quality also depended on the chuck velocity *V_u_*. The use of a higher chuck velocity resulted in lower roughness *Ra*. On the other hand, the use of a velocity of *V_u_* = 10 mm/s (for any forming angle *α*) led to a lower surface quality of the rolled parts. The use of a reduction ratio of *δ* = 1.13 resulted in higher roughness *Ra*. An increase in the reduction ratio value led to improved surface quality. In most of the rolling variants studied, an increased cross-sectional reduction in the samples improved the surface quality. This can be seen in the charts presented in Figure 5 and Figure 6, where the obtained results of roughness *Ra* with varying reduction ratios from *δ* = 1.13 to 1.53 are presented.

The analysis of the process shows that the setting of the angle *θ* of the rollers to the rolling axis significantly affects the surface quality. This angle is important for the resultant velocity of the rolled material. The shaped product is initially moved axially due to the askew positioning of the tapered rollers to the rolling axis. In addition, the process uses a jaw chuck (Figure 2, item no. 7), in which one end of the workpiece is placed. Using an axial cylinder (Figure 2, item no. 8), it is possible to regulate the velocity of movement of the rolled product. This affects the quality of the products (surface, defects) and also the efficiency of the process.

The resultant axial velocity of the rollers (resulting askew positioning of the working tools to the rolling axis at an angle *θ*), which is uncorrelated with the velocity of the chuck *V_u_*, causes visible helical spiral grooves on the circumference of the forging. A higher tool angle (e.g., *θ* = 7.5) relative to a low *V_u_* speed (e.g., 10 mm/s) will result in the workpiece being pushed through the tools rather than being pulled through the chuck. This results in a dense number of grooves on the surface (a similar effect is shown in Figure 4a). In extreme cases, buckling of the shaped workpiece can occur.

### 3.2. Numerical Calculations

Based on the surface roughness results, the resulting dataset was divided into three equal intervals: 0–6; 6.1–14 and >14 µm. The intervals were assigned the names “0”, “1” and “2”. This was done to avoid an unbalanced dataset, which would affect the results of the machine learning models, as well as creating the need to use additional libraries to help compensate for these discrepancies.

The numerical results made it possible to estimate the performance of the selected classification models. Table 3 presents the accuracy of each model as well as the hyperparameters at which these results are obtained. Their selection was carried out using the BayesSearch function. A three-fold cross-validation was performed in order to maximise the effectiveness of the models. Other metrics, such as precision, recall and F1-score are presented in Table 4, which are given for each of the three classes (0, 1, 2).

The results demonstrate that random forest and logistic regression yield high accuracies. For both classification models, the values were the same and equal to 0.93. The XGBoost model yielded an accuracy of 0.86, while the SVC had the lowest accuracy value of 0.71. It should be mentioned that the logistic regression model made use of three solvers: liblinear, newton-cg and saga. Based on data from the BayesSearch library, the liblinear solver was found to provide the most accurate prediction of material roughness.

**Figure 7:**
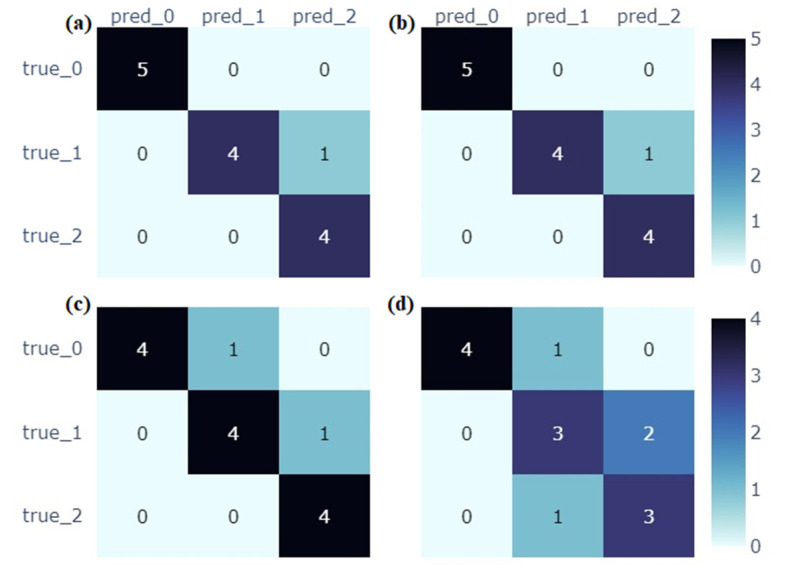
Confusion matrices for the following models: (**a**) random forest, (**b**) logistic regression, (**c**) XGBoost and (**d**) SVC.

An analysis of the results shows agreement between the random forest and regression logistic models. For class 0, all metrics, i.e., precision, recall and F1-score, are equal to 1, meaning that the models have identified this class accurately. For class 1, the precision and F1-score are high and amount to 1 and 0.89, respectively. The recall value is 0.8, which is due to the fact that the models omitted true cases for this class. This phenomenon is visible in Figure 7a,b, where the models predicted class 2, instead of class 1. For class 2, the precision was 0.8 and the recall was 1. This means that the models were characterised by false-positive classifications. Another tested model was XGBoost, which obtained a precision value of 1 and a recall value of 0.8 for class 0. This results from a misprediction made by the model, where it considered class 1 as one of the correct results (Figure 7c). The F1-score was 0.89, which was slightly lower than the values obtained by the previous two models. For classes 1 and 2, the precision reached a value of 0.8. These results are due to the prediction of false-positive classifications by the model. The F1-score value for the aforementioned classes was 0.8 and 0.89, respectively. These values are similar to those achieved by the random forest and logistic regression models. The overall accuracy of the XGBoost model is 0.86. This result is slightly lower than those obtained by the first two classification models.

The last model to be analysed is the Support Vector Classifier (SVC). A confusion matrix showing its performance is presented in Figure 7d. For class 0, the precision is 1 and the recall is 0.8. This yields an F1-score value of 0.89. This is due to one misprediction made by the model. For class 1, the precision and recall are the same and amount to 0.6. This is due to two misclassifications by the model. In contrast, for class 2, these metrics are equal to 0.6 (precision) and 0.75 (recall), respectively. The lower recall value results from one false-negative classification. Compared to the results obtained by the previous three models, these values are significantly lower. The overall model accuracy is 0.71, which is the lowest for all tested models. A similar observation was made for the F1-score of classes 1 and 2, with the values being 0.6 and 0.67, respectively.

**Figure 8:**
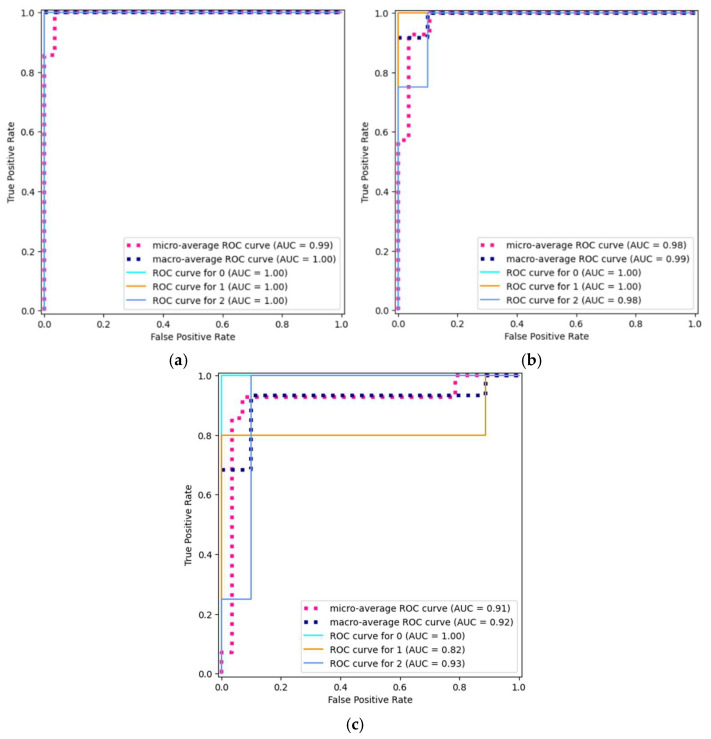
ROC curves for the following models: (**a**) random forest, (**b**) logistic regression and (**c**) XGBoost.

ROC curves were used to assess the performance of the applied models. The RocCurveDisplay method from the scikit-learn library was used to that end. The ROC curves are shown in Figure 8 for the three best classification models, i.e., random forest, logistic regression and XGBoost. The curves illustrate the relationships between the True Positive Rate and False Positive Rate for the individual classes. The results are shown for a multiclass classification problem by the One-vs.-Rest strategy. For the random forest model, the total area under the curve (AUC) is equal to 1. The ROC curves for all classes are almost perfect, showing that the model can perfectly distinguish between classes. The micro- and macro-average AUC values are also high, which indicates good generalisation of the results for the three classes. The logistic regression model yielded similar results to those obtained with random forest. The only difference was the AUC result for class 2. This model achieved a lower value of 0.98.

It should be stressed that the ROC curve illustrates how the classification of predictions made by a classifier model change at varying threshold values. Therefore, there are differences in the plots of random forest and logistic regression. For comparison, the results presented in the confusion matrices (Figure 7) relate to one fixed decision threshold. For the last model, i.e., XGBoost, the AUC value was equal to 1 for class 0, 0.82 for class 1, and 0.93 for class 2. A generalised measure of the model performance was also presented with the use of micro- and macro-average ROC curves. The former reached an AUC value of 0.91, while the latter obtained an AUC value of 0.92. As previously, its prediction accuracy is slightly lower compared to that of the other two models, i.e., random forest and logistic regression.

## 4. Conclusions

This publication presents results from experimental studies of the skew rolling process of steel forgings of C60 grade. Numerical calculations were also carried out using machine learning techniques using classification.

Based on the experimental results obtained, it was observed that an increase in both the forming angle *α* and the tool angle *θ* leads to increased surface roughness of C60 steel samples. In addition, an increase in the chuck velocity *V_u_* results in lower roughness Ra. The use of lower velocity values, such as 10 mm/s (for each forming angle *α*), has a negative effect on the quality of the rolled parts. An increase in the reduction ratio *δ* results in an improved surface quality of steel samples. It was determined that higher *V_u_* velocities should be used for reduction ratios lower than *δ* ≤ 1.13. For larger cross-sectional reduction (*δ* ≥ 1.3), a chuck velocity *V_u_* should be at least two times smaller than the resultant axial velocity of the roll. This is the result of the askew positioning of the working tools to the rolling axis at an angle *θ*. These setting correlations will ensure improved product quality.

The classification models, random forest and logistic regression, yield the highest accuracy of the results, with an average accuracy value of 0.93. A graphical analysis was carried out using the ROC curve, where the random forest classifier obtained the highest AUC value.

The numerical research carried out should be continued using a larger dataset. The obtained results could help prevent the formation of spiral grooves on rolled parts. Such grooves are formed in both the cylindrical and the tapered part of steel products. It should be added that machine learning techniques can also be useful in calculating force parameters, as well as in predicting possible internal cracks.

## Figures and Tables

**Table 1:** Chemical composition of C60-grade of steel.

**C**	**Mn**	**Si**	**P**	**S**	**Cr**	**Ni**	**Cu**	**Mo**
0.59	0.66	0.23	0.013	0.027	0.1	0.09	0.2	0.02
**V**	**Al**	**Ti**	**Sn**					
0.003	0.029	0.02	0.029					

**Table 2:** Experimental settings.

*α* (°)	*θ* (°)	*V_u_* (mm/s)	*δ* (-)
15 or 20 or 25	2.5	10	1.13
			1.3
			1.53
		20	1.13
			1.3
			1.53
		40	1.13
			1.3
			1.53
	5	10	1.13
			1.3
			1.53
		20	1.13
			1.3
			1.53
		40	1.13
			1.3
			1.53
	7.5	10	1.13
			1.3
			1.53
		20	1.13
			1.3
			1.53
		40	1.13
			1.3
			1.53

**Table 3:** Accuracy and hyperparameters for individual classification models.

Model	Accuracy	Hyperparameters
Random Forest	0.93	criterion = entropy, max_depth = 12
Logistic Regression	0.93	solver = liblinear, penalty = l2, max_iter = 10,000
XGBoost	0.86	objective = reg:logistic, n_estimators = 32
SVC	0.71	kernel = rbf, gamma = scale

**Table 4:** Metrics of individual classification models.

Class	Precision	Recall	F1-Score	Precision	Recall	F1-Score
	Random Forest			Logistic Regression		
**0**	1	1	1	1	1	1
**1**	1	0.8	0.89	1	0.8	0.89
**2**	0.8	1	0.89	0.8	1	0.89
	XGBoost			SVC		
**0**	1	0.8	0.89	1	0.8	0.89
**1**	0.8	0.8	0.8	0.6	0.6	0.6
**2**	0.8	1	0.89	0.6	0.75	0.67

## Data Availability

Data are contained within the article.

## References

[1] Contreras-Fortes J., Rodríguez-García M.I., Sales D.L., Sánchez-Miranda R., Almagro J.F., Turias I. (2024). A machine learning approach for modelling cold-rolling curves for various stainless steels. Materials.

[2] Zhao Y., Song Y., Li F., Yan X. (2023). Prediction of mechanical properties of cold rolled strip based on improved extreme random tree. J. Iron Steel Res. Int..

[3] Li W., Xie L., Zhao Y., Li Z., Wang W. (2020). Prediction model for mechanical properties of hot-rolled strips by deep learning. J. Iron Steel Res. Int..

[4] Song L., Xu D., Wang X., Yang Q., Ji Y. (2022). Application of machine learning to predict and diagnose for hot-rolled strip crown. Int. J. Adv. Manuf. Technol..

[5] Wang Z., Huang Y., Liu Y., Wang T. (2023). Prediction model of strip crown in hot rolling process based on machine learning and industrial data. Metals.

[6] Idzik C., Krämer A., Hirt G., Lohmar J. (2024). Coupling of an analytical rolling model and reinforcement learning to design pass schedules: Towards properties controlled hot rolling. J. Intell. Manuf..

[7] Zhao J., Li J., Qie H., Wang X., Shao J., Yang Q. (2023). Predicting flatness of strip tandem cold rolling using a general regression neural network optimized by differential evolution algorithm. Int. J. Adv. Manuf. Technol..

[8] Lian W., Du F., Pei Q. (2024). Prediction of rolling force during isothermal rolling process based on machine learning. Eng. Appl. Artif. Intell..

[9] Thakur S.K., Das A.K., Jha B.K. (2023). Application of machine learning methods for the prediction of roll force and torque during plate rolling of micro-alloyed steel. J. Alloys Metall. Syst..

[10] Bagheripoor M., Bisadi H. (2013). Application of artificial neural networks for the prediction of roll force and roll torque in hot strip rolling process. Appl. Math. Model..

[11] Trzepieciński T., Kubit A., Dzierwa A., Krasowski B., Jurczak W. (2021). Surface Finish Analysis in Single Point Incremental Sheet Forming of Rib-Stiffened 2024-T3 and 7075-T6 Alclad Aluminium Alloy Panels. Materials.

[12] Möllensiep D., Detering L., Kulessa P., Steinhof M., Kuhlenkötter B. (2024). Prediction of Forming Accuracy in Incremental Sheet Forming Using Artificial Neural Networks on Local Surface Representations. Int. J. Adv. Manuf. Technol..

[13] Li X., Fu Z., Shu J., Ji B., Ji B. (2024). A Modified Physics-Informed Neural Network to Fatigue Life Prediction of Deck-Rib Double-Side Welded Joints. Int. J. Fatigue.

[14] Pater Z., Tomczak J., Bulzak T., Walczuk-Gągała P. (2022). Numerical and experimental study on forming preforms in a CNC skew rolling mill. Arch. Civ. Mech. Eng..

[15] Pater Z., Tomczak J., Lis K., Bulzak T., Shu X. (2020). Forming of rail car axles in a CNC skew rolling mill. Arch. Civ. Mech. Eng..

[16] Xia Y., Shu X., Shi J., Wang Y., Pater Z., Wang J. (2022). Forming quality research on the variable-diameter section of the hollow axle in three-roll skew rolling. Materials.

[17] Zhang S., Shu X., Xia Y., Wang J. (2021). Formation mechanism and control of the spiral marks of three-roll skew-rolled hollow shafts. Metalurgija.

[18] Wang J., Shu X., Ye C., Li Z., Li S., Xu H., Wang Y., Deng Y., Chen Q. (2023). Study on forming quality of three-roll skew rolling hollow axle. Int. J. Adv. Manuf. Technol..

[19] Wang J., Shu X., Ye C., Zhang S., Li Z., Xu H., Wang Y., Deng Y., Li S. (2024). Study on fatigue performance of three-roll skew rolling hollow axle. J. Mater. Eng. Perform..

[20] Shu X., Zhang S., Shu C., Wang J., Ye C., Xia Y., Essa K., Pater Z. (2022). Research and prospect of flexible forming theory and technology of hollow shaft by three-roll skew rolling. Int. J. Adv. Manuf. Technol..

[21] Cao X., Wang B., Zhou J., Shen J., Lin L. (2021). Exploratory experiment and numerical simulation investigation on a novel flexible skew rolling of hollow shafts. Int. J. Adv. Manuf. Technol..

[22] Tomczak J., Pater Z., Bulzak T., Lis K., Kusiak T., Sumorek A., Buczaj M. (2021). Design and technological capabilities of a CNC skew rolling mill. Arch. Civ. Mech. Eng..

[23] scikit-learn. https://scikit-learn.org/stable/supervised_learning.html.

[24] dmlc XGBoost. https://xgboost.readthedocs.io/en/stable/.

